# Inducing I_to,f_ and phase 1 repolarization of the cardiac action potential with a Kv4.3/KChIP2.1 bicistronic transgene

**DOI:** 10.1016/j.yjmcc.2021.11.004

**Published:** 2022-03

**Authors:** Nan Wang, Eef Dries, Ewan D. Fowler, Stephen C. Harmer, Jules C. Hancox, Mark B. Cannell

**Affiliations:** School of Physiology, Pharmacology & Neuroscience, Faculty of Biomedical Sciences, University of Bristol, University Walk, Bristol BS8 1TD, United Kingdom

**Keywords:** Cardiac action potential, Transient outward current, Excitation-contraction coupling, Transgene expression, K channels, Heart failure

## Abstract

The fast transient outward potassium current (I_to,f_) plays a key role in phase 1 repolarization of the human cardiac action potential (AP) and its reduction in heart failure (HF) contributes to the loss of contractility. Therefore, restoring I_to,f_ might be beneficial for treating HF. The coding sequence of a P2A peptide was cloned, in frame, between Kv4.3 and KChIP2.1 genes and ribosomal skipping was confirmed by Western blotting. Typical I_to,f_ properties with slowed inactivation and accelerated recovery from inactivation due to the association of KChIP2.1 with Kv4.3 was seen in transfected HEK293 cells. Both bicistronic components trafficked to the plasmamembrane and in adenovirus transduced rabbit cardiomyocytes both t-tubular and sarcolemmal construct labelling appeared. The resulting current was similar to I_to,f_ seen in human ventricular cardiomyocytes and was 50% blocked at ~0.8 mmol/l 4-aminopyridine and increased ~30% by 5 μmol/l NS5806 (an I_to,f_ agonist). Variation in the density of the expressed I_to,f_, in rabbit cardiomyocytes recapitulated typical species-dependent variations in AP morphology. Simultaneous voltage recording and intracellular Ca^2+^ imaging showed that modification of phase 1 to a non-failing human phenotype improved the rate of rise and magnitude of the Ca^2+^ transient. I_to,f_ expression also reduced AP triangulation but did not affect I_Ca,L_ and I_Na_ magnitudes. This raises the possibility for a new gene-based therapeutic approach to HF based on selective phase 1 modification.

## Highlights

• Action potential phase 1 depends on fast transient outward current (I_to,f_).
• Construction of a bicistronic transgene for Kv4.3 and KChIP2.1 with P2A separator
• Expressed bicistronic Kv4.3/KChIP2.1 proteins traffic to the cell surface membrane
• Viral transduction with Kv4.3/KChIP2.1 increases I_to,f_ in cardiomyocytes.
• Kv4.3/KChIP2.1 transgene expression increased AP phase 1 and EC coupling

## Graphical abstract

**Figure:**
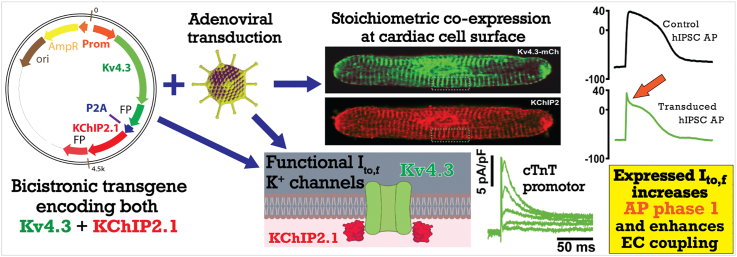

## 1 Introduction

Differences in outward K^+^ currents due to variations in K^+^ channel and/or accessory subunit(s) expression are primary determinants of species- and region-dependent variations in cardiac action potential (AP) morphology [[1], [2], [3]]. Differences in the (fast) transient outward potassium current (I_to,f_) contribute significantly to both interspecies and region-dependent variation in AP profile [1,3] and I_to,f_ underlies the early repolarization phase (phase 1) of the human cardiac AP [[4], [5], [6]]. In some species (such as rabbit [7]), the transient outward current I_to_ may include additional components such as a calcium-activated chloride current I_Ca(Ca)_ but this does not seem to be the case for human ventricle [4,8]. In human heart failure, loss of I_to,f_ contributes to loss of phase 1 of the AP [9] and is accompanied by down-regulation of the K^+^ channel β-subunit KChIP2 [10,11]. As the depth/rate of phase 1 repolarization decreases, dyssynchronous and reduced Ca^2+^ release from the sarcoplasmic reticulum (SR) develops [12,13] due to the reduction in triggering L-type Ca^2+^ current (I_Ca,L_) [12,14] and this contributes to the reduction in contractility although other cellular changes also play a role (for review see [15]).

Such results support the idea that modulation of I_to,f_ could be beneficial in heart failure [4]. We recently showed that electrophysiological restoration of phase 1 of the AP can improve SR release synchrony, suppress arrhythmogenic late Ca^2+^ spark production and increase the amplitude of the Ca^2+^ transient in a heart failure model [16] in agreement with the earlier demonstration that the loss of human phase 1 repolarization impairs excitation-contraction coupling (ECC) [14]. However, increasing I_to,f_ pharmacologically (with the I_to_ agonist NS5806) in a dog ventricular wedge preparation led to the emergence of Brugada-like electrical abnormalities [17] while in intact rabbit heart, NS5806 also promoted arrhythmias (although Brugada-like behaviour did not necessarily appear) and was suggested to be the consequence of defective Ca^2+^ cycling [18]. In dog, increasing phase 1 repolarization decreased Ca^2+^ entry, SR release and contraction [19]. However, the effects of NS5806 on the heart could be complicated by off-target effects such as Na^+^ current inhibition [20,21].

As an alternative to pharmacological I_to,f_ augmentation, I_to,f_ could be modulated by exogenous gene expression. KChIP2.1 is the predominant KChIP2 isoform in human heart [22] and when combined with Kv4.3 produces an I_to,f_ very similar to native human I_to,f_ [10]. While KChIP2.2 can also enhance Kv4.3 channel currents, slow inactivation and increase the rate of recovery, KChIP2.1 co-expression was more potent [23] and could potentially reverse heart failure-induced changes in these parameters. While both short and a long splice variants of Kv4.3 (Kv4.3S and Kv4.3 L respectively) are expressed in human ventricles [10,24], it appears that only the Kv4.3S isoform is reduced in heart failure [10]. A study on Kv4.3 isoforms expressed with KChIP2 suggested that specifically upregulating Kv4.3S could be more beneficial than a general Kv4.3 upregulation in terms of I_to_ density restoration in HF [25]. Therefore in this study, we constructed and expressed a bicistronic transgene for both Kv4.3S and KChIP2.1 incorporating a modified porcine teschovirus-1 ribosomal skipping sequence (P2A) [26] to examine how such a vector might recapulate I_to,f_ in cell systems to modify AP morphology and ECC.

## 2 Material and methods

All experiments were performed in accordance with the UK Home Office Animals (Scientific Procedures) Act 1986 and institutional approval by the University of Bristol ethics committee. Detailed methods for HEK and hiPSC-CM cell culture, Western blotting and viral vector production and transfection are provided in the SI appendix.

### 2.1 Bicistronic constructs

**Fig. 1:**
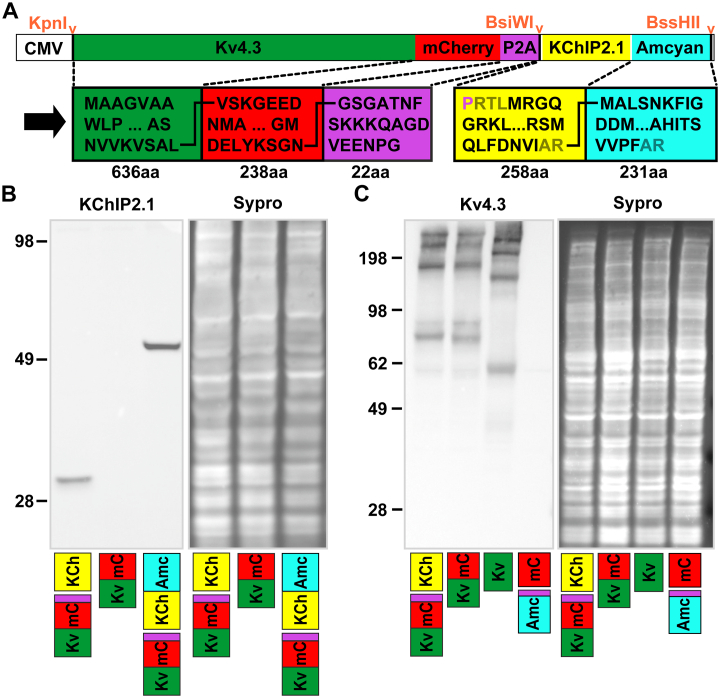
A. Plasmid vector for bicistronic for Kv4.3 and KChIP2.1 with amino acids shown in black (linkers in brown). A CMV or cardiac-specific troponin T promoter (cTnT) was used in different cell types. B. Western blot of KChIP2.1 expression by the bicistronic transgene in HEK293 cells. A single band corresponding to the estimated molecular weight of KChIP2.1 or KChIP2.1-Amcyan was detected in lysates from bicistronic transgene transfected cells but not from cells transfected with Kv4.3-mCherry alone. C Expression of Kv4.3-mCherry upstream of the P2A sequence was detected at an apparent molecular weight of ~80 kDa while expression of Kv4.3 alone was detected at ~60 kDa. These data are consistent with the expected weights of Kv4.3 (71 kDa) and mCherry (20 kDa), given a − 20% gel shift for K channel protein [61]. Additional higher molecular weight bands possibly reflect multimeric forms of Kv4.3. Lysates of cells transfected with untagged Kv4.3 and Amcyan-P2A-mCherry were used as positive and negative controls respectively and SYPRO® staining was used as internal loading control. (For interpretation of the references to colour in this figure legend, the reader is referred to the web version of this article.)

The cDNA constructs encoding human short Kv4.3 isoform 1 precursor (*KCND3* gene, NCBI reference sequence NM_172198.2) and KChIP2.1 (*KCNIP2* gene, NCBI reference sequence NM_173192.2) were synthesized and sequenced by GenScript (GenScript, Piscataway, New Jersey, USA). A red fluorescent reporter mCherry followed by the coding sequence of the self-cleaving P2A peptide (with GSG amino acids at the N-terminus [26]) was PCR amplified from a bicistronic vector (Plasmid #45350, Addgene, Watertown,Massachusetts, USA) and fused to the C-terminus of Kv4.3 in a pcDNA3.1^+^ backbone vector. *Bsi*WI and *Bss*HII digestion sites were simultaneously added to the 3′-end of the P2A sequence allowing addition of KChIP2.1 or KChIP2.1-Amcyan into the second cistron as needed (see Fig. 1A). Methods for recombinant adenovirus construction, transfection and Western blotting are given in the supplement.

### 2.2 Adult rabbit ventricular myocyte isolation, culture and adenovirus transfection

Rabbit ventricular myocytes were selected due to their similar morphology and electrical properties to human myocytes and since they have an intrinsically low I_to,f_ density they provide a model in which I_to,f_ can be increased by our bicistronic gene expression to human levels (or even higher). LV cardiomyocytes were enzymatically isolated from the hearts of adult New Zealand rabbits with an established method [14]. Rabbits were killed by lethal injection of sodium pentabarbitone (i.v. 150 mg/kg) in accordance with the University Animal Ethics Committee guidelines. The heart was rapidly removed and mounted on a Langendorff perfusion apparatus for enzymatic dissociation. Initially, the heart was perfused with a Ca^2+^-free modified Tyrode solution containing (in mmol/l): 137 NaCl, 4 KCl, 1 MgCl2, 10 HEPES, 10 glucose, pH 7.4 (with NaOH) for approximately 5 min. Ventricular myocytes were then enzymatically dissociated by perfusing the heart, for 10–13 min, with the modified Tyrode solution to which the following were added: collagenase (Worthington; 1.0 mg/ml), protease (Sigma Type I; 0.1 mg/ml) and 0.2 mmol/l CaCl_2_.

For culture, myocytes were first suspended in M199 supplemented with 5 mmol/l taurine, 5 mmol/l creatine, 0.01 mmol/l ascorbic acid, 25 mmol/l HEPES, 0.2% bovine serum albumin, 10 U/ml penicillin and 10 μg/ml streptomycin (Sigma Aldrich) and gassed with carbogen. After 2-h incubation at 37 °C cardiomyocytes were harvested and plated on glass coverslips coated with laminin (Sigma Aldrich) for 3 h followed by transduction with adenovirus at multiplicity of infection (MOI) from 5 to 20. Myocytes were patch-clamped or immunostained 40 h after viral exposure.

### 2.3 Cultured cells

More detailed methods for HEK293 and hiPSC-CM experiments are given in the supplement. During electrical recording, HEK293 cells were bathed in extracellular solution containing (in mmol/l): 126 NaCl, 5.4 KCl, 1 MgCl_2_, 2 CaCl_2_, 10 HEPES, 11 glucose and pH adjusted to 7.4 with NaOH. The pipette solution contained (in mmol/l): 125 KAspartate, 10 KCl, 1 MgCl_2_, 10 NaCl, 5 MgATP, 10 HEPES, and pH adjusted to 7.2 with KOH. For I_to,f_ recordings, 200 μM CdCl_2_ was applied. After a brief step from a holding potential of −80 mV to −40 mV to inactivate sodium channels, 500-ms voltage steps from −30 to +50 mV were used to elicit I_to_ currents in hiPSC-CMs.

### 2.4 Electrophysiology

For I_to,f_ recordings from ventricular myocytes, 200 μM CdCl_2_ was applied extracellularly to inhibit L-type Ca^2+^ current [27] and Ca^2+^ − activated Cl^−^ current [7]. I_to,f_ currents were elicited by 500 ms voltage steps from −30 to +40 mV in 10 mV increments following an initial depolarization from a holding potential of −70 mV to −40 mV that inactivated Na^+^ currents. To examine the kinetics of the I_to,f_ recovery from inactivation, myocytes were held at −70 ms for various interpulse intervals ranging from 5 to 90 ms. Whole-cell currents were recorded at 35 °C and digitized at 10 kHz (after a 5 kHz cut-off Bessel filter). APs were elicited at 1 Hz by 4-ms suprathreshold current stimuli. Resting membrane potential (RMP) was measured immediately before the AP upstroke. AP amplitude (APA), AP plateau potential (at 20 ms after the initiation of the action potential upstroke), and AP durations at 20% and 90% repolarisation (APD20 and APD90, respectively) were measured.

Whole-cell Na^+^ currents (I_Na_) were recorded at room temperature with sampling frequency of 50 kHz. Extracellular solution was composed of (in mmol/l): 130 CsCl, 1.2 MgCl_2_, 20 HEPES, 5 NaCl, 1 CaCl_2_, 11 glucose, 0.02 Nifedipine and pH adjusted with CsOH to 7.4. Intracellular solution consisted of (in mmol/l): CsCl 130, MgCl_2_ 0.4, NaCl 5, HEPES 10, glucose 5, EGTA 5, MgATP 5 with pH adjusted to 7.2 with CsOH. Series resistances (2–4 MΩ) were electronically compensated by up to 60%. All reagents to prepare solutions were purchased from Sigma Aldrich or ThermoFisher Scientific (Paisley, UK) unless otherwise indicated.

Action potentials from cultured cardiomyocytes were elicited in current-clamp mode, by 4-ms suprathreshold depolarizing current pulses at 1 Hz. Action potential duration (APD) was quantified at 20%, 30% and 90% repolarization. Short-term variability of APD at 90% repolarization was determined using a sliding window of 10–14 consecutive beats using Σ(|APDi+1 -APDi|)/[nbeats×√2].

### 2.5 Immunostaining, confocal imaging and analysis

HEK293 cells and transduced myocytes were fixed in 2% paraformaldehyde for 10 min, permeabilized with 0.2% Tx-100 for 15 min and blocked with 3% bovine serum albumin (Invitrogen) and 10% goat serum (ThermoFisher Scientifics). Samples were then labelled with primary antibodies including anti-Caveolin 3 (anti-Cav3; BD Bioscience, San Jose, USA; 1/500 dilution) anti-KChIP2 (Alomone, Israel; 1/500 dilution) overnight at 4 °C. Western blots showed this antibody recognised our construct with high specificity. Specificity controls for KChIP2 labelling in transduced myocytes were performed by pre-incubating the anti-KChIP2 antibody with an immunizing peptide (Alomone) comprised of the amino acid sequences corresponding to the KChIP2 epitope at a 1:10 ratio (wt:wt) overnight at 4 °C (see Fig. S2A,B). Secondary antibodies (KChIP2.1: goat anti-rabbit IgG Alexa 488, 1/400; Cav3: goat anti-mouse IgG Alexa 647, 1/400, Invitrogen Molecular Probes) were applied for 1 h at room temperature. Labelled cells were imaged with Zeiss 880 confocal microscope using a 63× oil immersion objective. Fluorophores were excited at 488 nm (Alexa488), 594 nm (mCherry), and 633 nm (Alexa647). The degree of colocalization of Kv4.3 and KChIP2 was quantified using the unbiased Pearson's correlation coefficient. Masks capturing the regions of sarcolemma and T-tubules labelling were constructed from the Cav3 labelling.

### 2.6 Statistical analysis

Data are presented as mean ± SEM. n/N depicts of n individual and independent myocyte experiments from N animals. Statistical comparisons employed paired or unpaired *t*-test, one-way ANOVA (with Bonferroni post hoc test) and hierachial tests across groups as appropriate. *p* < 0.05 was taken as the limit of statistical confidence.

## 3 Results

### 3.1 Bicistronic expression of Kv4.3 and KChIP2.1 in HEK293 cells

Fig. 1A shows the general organization of the bicistronic vector designed for producing I_to,f_ . The human Kv4.3-mCherry and KChIP2.1 sequences were separated by a P2A sequence and Amcyan was added to KChIP2.1 when separate detection of this component was needed. The last glycyl-prolyl peptide bond of the P2A peptide (shown in purple) should be skipped by ribosomes during translation and incorporated into KChIP2.1. Fig. 1B confirms that KChIP2.1 was expressed as a seperate protein in HEK293 cells. When Amcyan was added to KChIP2.1, the detected molecular weight increased as expected. Fig. 1C shows that identical bands above ~70 kDa were detected by an anti-Kv4.3 antibody in lysates prepared from cells overexpressing Kv4.3-mCherry-P2A-KChIP2.1 and Kv4.3-mCherry alone. This shows that the P2A insert resulted in efficient cleavage of the whole construct into separate Kv4.3 and KChIP2.1 proteins. Note that the ion channel protein was routinely found as high molecular weight aggregates (in addition to the monomer form) due to hydrophobic interactions between N-termini of Kv4.3 subunits [28].

### 3.2 Characterization of I_to,f_ currents in HEK293 cells expressing bicistronic Kv4.3/KChIP2.1

**Fig. 2:**
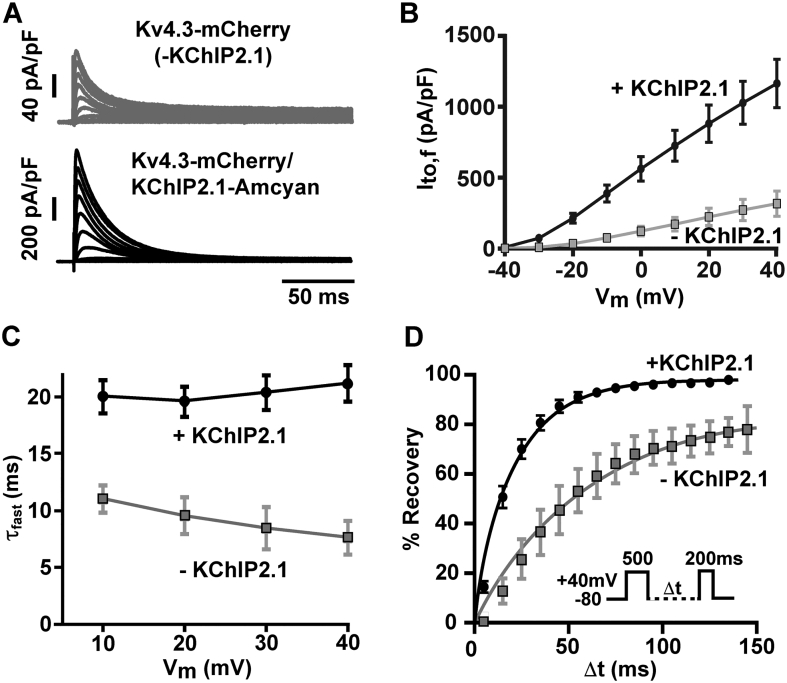
Biophysical properties of I_to,f_ currents produced by Kv4.3 alone (-KChIP2.1) and with KChIP2.1 added via the bicistronic vector in HEK293 cells. A. Exemplar whole-cell currents elicited by 500 ms-depolarization pulses from −60 mV to +40 mV in 10 mV increments from a holding potential of −80 mV. B. The peak current at all potentials was increased by bicistronic addition of KChIP2.1 and the current activated at ~ − 30 mV as expected for Kv4.3 in the presence of KChIP2.1. C. The fast time constant of I_to,f_ inactivation as a function of test potential (Kv4.3 alone: 7.66 ± 1.5 ms, *n* = 5 vs. Bicistronic Kv4.3/KChIP2.1: 21.2 ± 1.6 ms, *n* = 18 at +40 mV; *p* < 0.002 Students *t*-test). D. Time-dependent recovery from inactivation at −80 mV. To calculate the recovery rate from inactivation, normalised tail peak current amplitudes were recorded at +40 mV and plotted as a function of the inter-pulse interval (Δt) (protocol illustrated in the inset). Error bars show mean ± s.e.m.

As illustrated in Fig. 2A, I_to,f_ currents recorded from Kv4.3 and bicistronic Kv4.3/KChIP2.1 transfected HEK293 cells exhibited a current-voltage relation (Fig. 2B) similar to that previously described for Kv4.3 and Kv4.3/KChIP2.1 channel complexes [23,29]. The time constant of the fast I_to,f_ inactivation component (τ_fast_) was largely independent of membrane potential and was ~2.5× faster than in the absence of KChIP2.1 co-expression (Fig. 2C), confirming that KChIP must have been expressed and trafficked to the surface membrane. The time-dependent recovery of I_to,f_ from inactivation at a holding potential of −80 mV was evaluated by a two-pulse protocol with increasing interpulse intervals (inset Fig. 2D). The recovery from inactivation followed a mono-exponential time course with a time constant of 20.4 ± 0.7 ms (*n* = 15). Consistent with previous reports [22,23], the recovery time constant of I_to,f_ produced by the bicistronic construct was faster than seen for Kv4.3-mCherry alone (Fig. 2D), further confirming successful KChIP2.1 expression and Kv4.3 targeting. To examine the biophysical properties of I_to,f_ in cells expressing excess (i.e. non-stoichiometric) KChIP2.1, we also co-transfected HEK293 cells with both bicistronic Kv4.3-mCherry/KChIP2.1 and KChIP2.1-Amcyan at a plasmid ratio of 1:1. The additional KChIP2.1 expression did not affect the peak amplitudes (*p* > 0.27, unpaired *t*-test), inactivation kinetics (*p* > 0.72, unpaired t-test) or the inactivation recovery time course (*p* > 0.9, unpaired t-test) of I_to,f_ (see supplementary Fig. S1A, S1B and supplementary Table S1). That additional KChIP2.1 expression did not affect I_to,f_ current properties suggests that the bicistronic construct produced the maximal effect of KChIP2.1 on Kv4.3.

### 3.3 Subcellular localization of the expressed I_to,f_ proteins

**Fig. 3:**
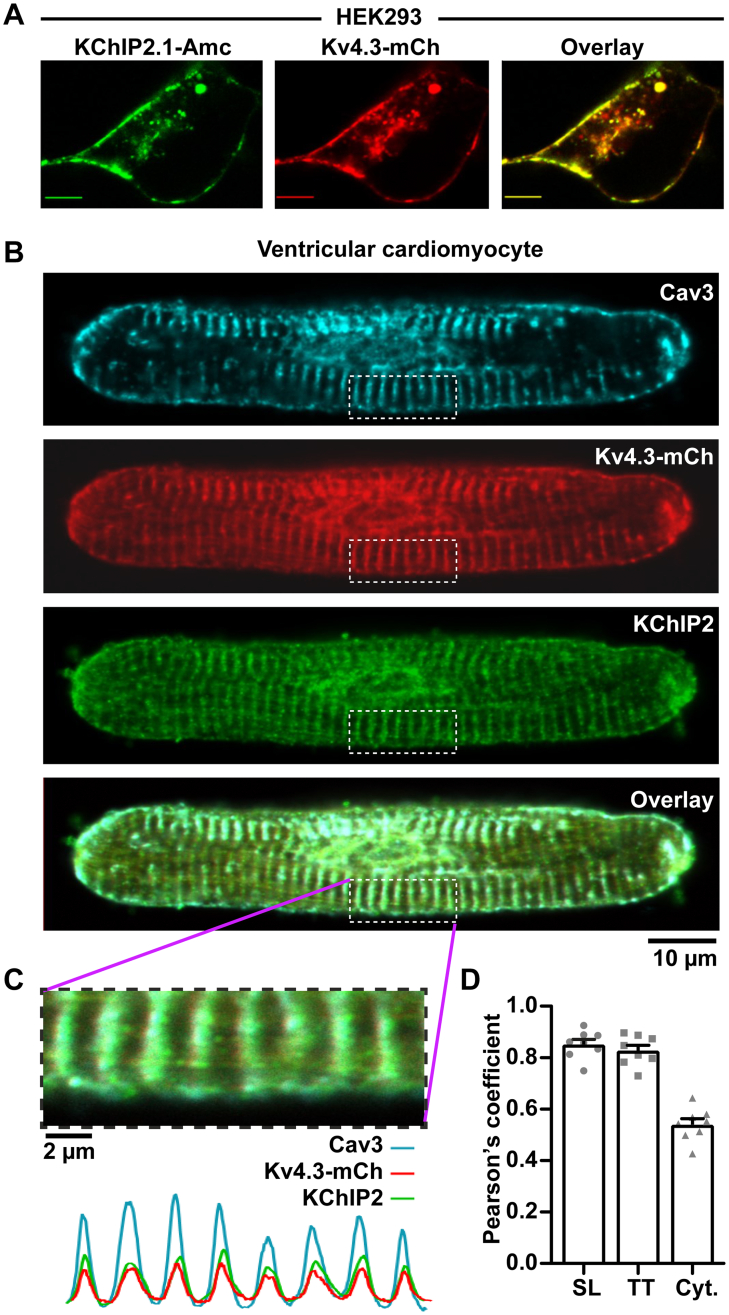
Subcellular colocalization of Kv4.3 and KChIP2.1. A. Exemplar confocal images of living HEK293 cells expressing bicistronic Kv4.3-mCherry and KChIP2.1-AmCyan. B. Representative confocal images of a transduced rabbit ventricular cardiomyocytes expressing Kv4.3-mCherry and KChIP2 (see also Fig. S2A). Surface membranes were labelled with an anti-Cav3 antibody (top). C shows an enlarged view of the boxed regions in B and the fluorescence intensity profile from each label at bottom. D Co-localization between Kv4.3 and KChIP2.1 in different subcellular compartments (sarcolemma -SL; t-tubules TT; and remaining cytoplasm -Cyt.) measured by unbiased Pearson's correlation coefficient (n/*N* = 8/3). Error bars show s.e.m.

Fig. 3A shows a typical HEK293 cell expressing fluorescently tagged Kv4.3 and KChIP2.1 proteins after transfection with the bicistronic construct. Both subunits strongly co-localized with a clear cell surface distribution. This observation was supported by a large Pearson's coefficient for colocalization of 0.874 ± 0.024 (Pearson's coefficient after using Costes' randomization was 0.0011 ± 0.0003, *p* < 0.0001, *n* = 8).

Adenovirus-mediated transduction of isolated rabbit ventricular myocytes with the Kv4.3-mCherry/KChIP2.1 vector also resulted in successful trafficking of the gene products to the surface membrane as shown by immuno-labelling and fluorecence imaging. Fig. 3B shows representative confocal images of a cultured myocyte after gene transduction. Exogenous Kv4.3 was reported by the red fluorescent protein tag (mCherry) while expression of KChIP2.1 in the transduced myocytes was detected by an anti-KChIP2 antibody. Strong Kv4.3 and KChIP2.1 labelling occured in and around T-tubular (TT) regions as identified by the caveolin-3 (Cav3) labelling (see Fig. 3C) as well as some perinuclear staining (see Also Fig. S2A). Analysis of the labelling across z-lines (Fig. 3C) showed that the expressed Kv4.3 was strongly aligned with KChIP2.1 labelling along TTs. By constructing a mask from the Cav3 labelling, we analyzed regional colocalization of Kv4.3 and KChIP2.1 in TTs and the surface sarcolemma (SL). As summarized in Fig. 3D, Pearson's correlation coefficients shows that a significant portion of the Kv4.3 and KChIP2 colocalized at the SL and TT with a relatively weaker colocalization outside these regions. As shown in HEK293 cells, Western blot analysis of transduced cardiomyocytes confirmed complete cleavage of the bicistronic construct with KChIP2.1 appearing as a separate isolated protein band (supplementary Fig. S2B). Collectively, these findings show that the bicistronic transgenes were successfully co-expressed in cardiomyocytes with separate Kv4.3 and KChIP2.1 proteins subsequently trafficking efficiently to the SL to generate a functional I_to,f_ (see below).

### 3.4 Characterization of the transgene-encoded channel currents in rabbit ventricular myocytes

**Fig. 4:**
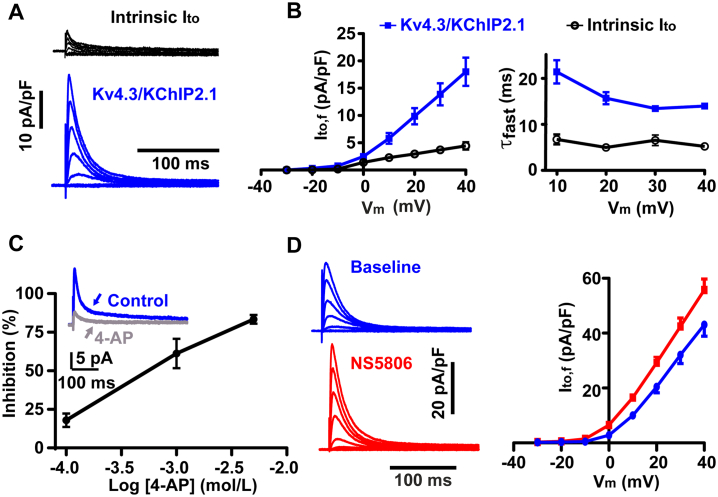
Characterization of I_to,f_ currents encoded by the bicistronic Kv4.3/KChIP2.1construct in rabbit ventricular cardiomycytes. A. Typical patch-clamp recordings of I_to_ currents from freshly isolated cardiomyocytes and 40 h after transduction. Currents were evoked by step depolarizations to potentials between −30 and + 40 mV (in 10 mV increments). B. The left panel shows current-voltage relationships of intrinsic I_to_ (n/*N* = 7/3) and exogenous I_to,f_ (n/*N* = 16/8) and the fast time constant of I_to,f_ inactivation (right hand panel). C. Concentration-dependence of the I_to,f_ block by 4-AP block (n/N = 7/4). Exemplar recordings of the I_to,f_ currents at +30 mV before and after application of 1 mmol/l of 4-AP are shown in the inset. D. Exemplar I_to,f_ traces recorded from a transduced myocyte under basal conditions and in the presence of NS5806 (5 μmol/l). The right hand panel shows the augmentation of the mean peak I_to,f_ current-voltage relationship by 5 μmol/l NS5806 (*p* < 0.001, n/N = 7/3, matched pair t-test). The cells used for these experiments were cultured for 48 h (40 h in A,B) to further increase peak I_to,f_ to minimise the contribution from NS5806-augmented native I_to_ (typically 3 pA/pF [21]). Error bars show mean ± s.e.m.

To characterize the function of channels assembled by the bicistronic construct, we performed whole-cell recordings in rabbit ventricular myocytes after adenoviral transduction in vitro*.* Adenoviral transfection with red fluorescent protein (RFP) without I_to,f_ components had no effect on the cell APD or I_Ca,L_ (Fig. S2C), but the bicistronic Kv4.3/KChiP2.1 construct produced a robust I_to,f_ compared to the low amplitude intrinsic I_to_ seen in freshly isolated epicardial ventricular myocytes (Fig. 4A). After 40 h in culture, the peak current density in transduced myocytes was 18.0 ± 2.6 pA/pF at +40 mV, which was approxiately 4 times larger than the endogenous I_to_ (Fig. 4B and Table 1). Exogenous I_to,f_ expression after 40 h of culture did not change the sustained (non-inactivating) outward current seen at the end of a 500 ms depolarizing pulse to +40 mV (see Table 1). This outward current was also present in freshly isolated myocytes (Table 1) suggesting that it was not related to I_to,f_ or the time in culture. I_to,f_ fast inactivation (τ_fast_) was approximately 2.5 times slower in transduced cells than the native I_to_ at positive V_m_ (Fig. 4B). It is notable that τ_fast_ at +40 mV (Table 1) was similar to that reported for human left ventricle (LV) sub-epicardial myocytes I_to,f_ (12.5 ± 0.8 ms -see [30]). I_to,f_ in transduced myocytes exhibited rapid mono-exponential recovery from inactivation with a time constant of 19.6 ± 0.9 ms (supplementary Fig. S1C) which was, again, similar to human I_to,f_ (~17 ms see Fig. 2B in [30]) and much faster than the smaller native I_to_ (2417 ± 117 ms [21]). To confirm the functional identity of the expressed I_to,f_, we applied 4-Aminopyridine (4-AP), a known inhibitor of I_to,f_ [6]. Fig. 4C shows that 4-AP inhibited 50% of I_to,f_ at approximately 0.8 mmol/l, similar to the Kd of 0.9 ± 0.07 mmol/l reported for Kv4 channels [31]. We also examined the response of the expressed I_to,f_ to NS5806, a compound that acts as an agonist for I_to,f_ current produced by Kv4.3/KChIP2 complexes [32,33]. Fig. 4Di illustrates the typical response to NS5806 (5 μmol/l). The drug produced a significant increase in peak currents over the entire activation being, on average, 32% larger at +30 mV, in good agreement with the reported dose-response relation for NS5806 in CHO-K1 cells [29] and the ~80% agonism at 10 μmol/l in dog epicardial cells [32].

**Table 1:** Electrophysiological effects of culture and viral bicistronic transduction in cardiomyocytes.

	Day 0, Freshly isolated	After 40-h culture, untransduced (P vs day 0)	After 40-h culture, Kv4.3/KChIP2.1 transduced	P-value untransduced vs transduced (both at 40h)
I_to,f_ amplitude @ +40 mV (pA/pF)	4.4 ± 0.7 (n/N = 7/3)	3.2 ± 0.4^ns^ (n/*N* = 6/3; *P* > 0.999)	18.0 ± 2.6** (n/N = 16/8)	0.0012
I_to,f_ decay τ_fast_ @ +40 mV (ms)	5.2 ± 0.5 (n/N = 7/3)	6.7 ± 0.9^ns^ (n/N = 6/3; *P* = 0.47)	14.0 ± 0.5^⁎⁎⁎^ (n/N = 16/8)	<0.001
Non-inactivating outward current @ +40 mV (pA/pF)	1.73 ± 0.13 (n/N 7/3)	1.9 ± 0.2^ns^ (n/N = 6/3; P > 0.999)	2.3 ± 0.2^ns^ (n/N = 16/8)	0.68
APD20 (ms)	95.5 ± 14.1 (n/*N* = 9/4)	83.1 ± 7.2^ns^ (n/*N* = 13/6; *P* = 0.81)	5.9 ± 1.1^⁎⁎⁎^ (n/N = 16/7)	<0.001
APD30 (ms)	157.2 ± 15.9 (n/*N* = 9/4)	136.1 ± 9.0^ns^ (n/N = 13/6; *P* = 0.45)	34.90 ± 5.6^⁎⁎⁎^ (n/N = 16/7)	<0.001
APD90 (ms)	281.8 ± 15.5 (n/N = 9/4)	321.6 ± 15.9^ns^ (n/N = 13/6; *P* = 0.15)	186.3 ± 8.0^⁎⁎⁎^ (n/N = 16/7)	<0.001
Triangulation (APD90-APD30; ms)	124.5 ± 10.7 (n/N = 9/4)	185.5 ± 9.8^⁎⁎⁎^ (n/N = 13/6; *P* < 0.001)	151.4 ± 5.3^⁎⁎^ (n/N = 16/7)	0.002
Short-term variability (ms)	5.3 ± 1.9 (n/N = 9/4)	7.1 ± 0.6^ns^ (n/N = 11/6; *P* = 0.121)	4.6 ± 0.4^⁎⁎^ (n/*N* = 10/6)	0.009
I_Ca,L_ at +10 mV (pA/pF)	-14.1 ± 1.4 (n/*N* = 14/4)	−9.65 ± 0.52* (n/N = 9/4; *P* = 0.031)	−11.35 ± 1.42^ns^ (n/N = 7/3)	>0.999
Ca^2+^ transient, rate of rise (τ_rise_; ms)	16.3 ± 1.0 (n/N = 12/6)	31.1 ± 3.9** (n/*N* = 17/7, *P* = 0.0013)	14.4 ± 1.2*** (n/*N* = 19/7)	<0.001
Ca^2+^ transient, amplitude (F/Fo)	2.17 ± 0.10 (n/N = 12/6)	1.37 ± 0.04*** (n/N = 17/7, P < 0.001)	1.55 ± 0.04* (n/N = 19/7)	0.031
Cell-shortening	4.9 ± 0.6% (n/*N* = 18/4)	3.4 ± 0.3% ^ns^ (n/*N* = 28/3; *P* = 0.06)	5.4 ± 0.4%^⁎⁎^ (n/*N* = 31/3)	0.0015
Whole-cell capacitance (pF)	181.5 ± 14.1 (n/N = 13/3)	139.0 ± 11.0* (n/N = 13/6; *P* = 0.04)	141.5 ± 8.8^ns^ (n/N = 13/6)	>0.999

*P*-values from one-way ANOVA; ns > 0.05, * *P* < 0.05, *P* < 0.01 and *** *P* < 0.001. Values are mean ± s.e.m.

### 3.5 Expressed I_to,f_ changes AP morphology and ECC in cardiomyocytes

**Fig. 5:**
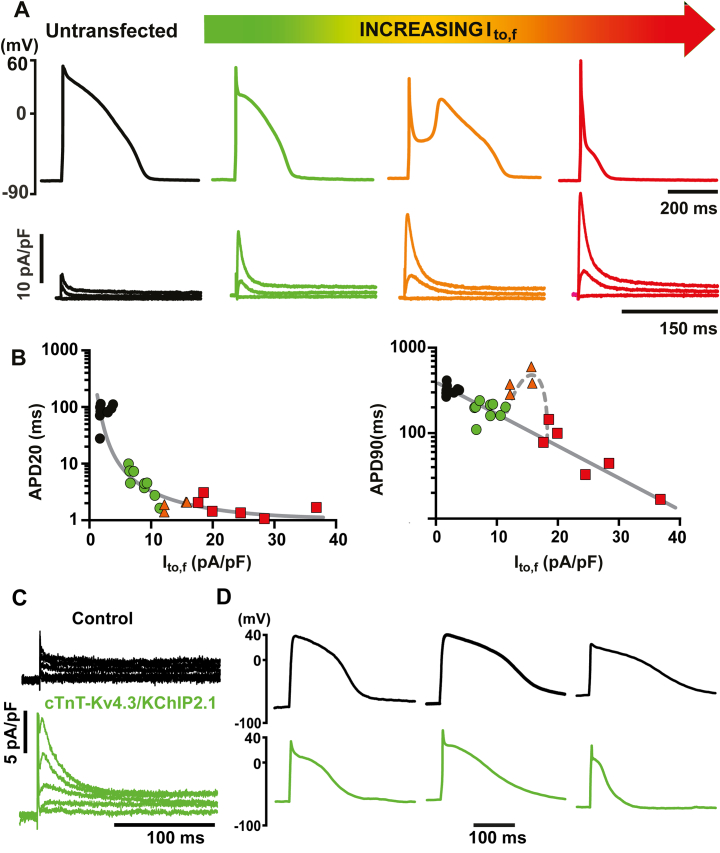
Increasing I_to,f_ density with bicistronic Kv4.3/KChIP2.1 expression affects cardiomyocyte AP waveform. Panel A shows APs recorded from 2 day cultured (untransduced black), and transduced myocytes with increasing I_to,f_ current densities at a cycle length of 1 s. Introduction of I_to,f_ (lower panel traces show exemplar records for pulses to −30, +10 and + 40 mV) resulted in a prominent AP phase 1 in all transduced myocytes. As I_to,f_ density increased, there was a progressive change in AP morphology that caused the AP to resemble that recorded in other species. B. Increasing density of I_to,f_ (at +40 mV) decreased APD20. APD90 (right panel) decreased approximately exponentially (solid line) with increasing I_to,f_ except when I_to,f_ was between ~12 and ~ 17 pA/pF (deviation highlighted as dashed line) where emergence of a marked ‘spike-and-dome’ morphology developed (as shown center right in A). All trend curves drawn by eye. C. Representative traces of I_to,f_ recorded in untransduced (Control) iPSC-CMs and iPSC-CMs expressing bicistronic Kv4.3-mCherry/KChIP2.1 under a cTnT promoter. After a brief step to −40 mV to inactivate Na^+^ channels, subsequent membrane depolarizations to −30 mV, −10 mV, +10 mV, +30 mV and + 50 mV were used to elicit I_to_ in control and transduced iPSC-CMs. D. Three examplar AP waveforms of iPSC-CMs without (black traces) or with expressed I_to,f_ currents (green lower traces). I_to,f_ densities in the exemplar transduced iPSC-CMs were 5–10 pA/pF at +40 mV. APs were elicited at a frequency of 1 Hz. (For interpretation of the references to colour in this figure legend, the reader is referred to the web version of this article.)

The transduced I_to,f_ density depends on the adenovirus titer and/or transduction efficiency which, with post hoc analysis, revealed the relationship between I_to,f_ density and AP configuration. Fig. 5A (left) shows an electrically-evoked AP waveform in control (untransduced) ventricular cardiomyocytes (upper panel) and the corresponding magnitude of the native I_to_ (bottom trace). The AP of untreated cells featured a small phase 1 notch and a high plateau potential. After transduction, an increasing I_to,f_ current (from 6 to 28 pA/pF) caused the depth of phase 1 to progressively increase with marked changes in overall AP morphology. In untransduced myocytes, the average early plateau potential (20 ms after the start of the AP) was 42.0 ± 1.6 mV (*n* = 17) and this was reduced to 23.4 ± 1.7 mV (*n* = 19) in transduced myocytes (*p* ≤ 0.0001, nested *t*-test) (see Fig. S2F). A moderate I_to,f_ density between 6 and 11 pA/pF (which is close to the reported human LV epicardial I_to,f_ density of 12.8 ± 0.6 pA/pF [30]) resulted in a phase 1 notch and early plateau potential similar to that seen in non-failing human cardiomyocytes (see e.g. Fig. 5 in [34]). The phase 1 notch further deepened with increasing I_to,f_ expression so that with current densities between 12 and 16 pA/pF the AP adopted a distinct canine-like ‘spike-and-dome’ AP morphology (orange traces). I_to,f_ currents density greater than ~18 pA/pF shortened the AP into a triangular shape as is typically seen in rodent myocytes (red traces) which have native I_to_ densities of 15–30 pA/pF [35]. The effect of increasing I_to,f_ density on AP durations at both 20% (APD20) and 90% repolarization (APD90) is summarized in Fig. 5B; there was a general trend toward AP shortening except for current densities between ~12 pA/pF and 16 pA/pF where APD90 tended to increase again. The kinetics of the fast inactivating component of the exogenous bicistronic I_to,f_ did not change with increasing I_to,f_ density (supplementary Fig. S2D) and was ~14 ms (at +40 mV). Since this is very similar to the time constant of native human I_to,f_ (~13 ms) [30] these data support the idea that our bistronic construct could be used to enhance the Kv4.3S/KChIP2.1 component of native I_to_ which is reduced in failure [10].

Triangulation of the AP measured as the difference in time to between 30% and 90% AP repolarisation (APD90–30) has been implicated as a serious pro-arrhythmogenic factor [36]. We found that APD90–30 as well as beat-to-beat variability in APD90 at 1 Hz became greater in cultured, untransduced myocytes as a result of remodelling in culture (as compared to freshly isolated cardiomyocytes) wherease in transduced cardiomyocytes, moderate levels of I_to,f_ expression (6–11 pA/pF) successfully reduced APD90–30 and beat-to-beat variability (summarized in Table 1). The latter is consistent with the proposed influence of I_to_ on AP variability [37].

We also examined whether expressing our transgenes under the control of the cardiac-specific cTnT promoter could produce a native I_to,f_ in human induced pluripotent stem cell-derived cardiomyocytes (iPSC-CMs). The AP waveforms of untransfected iPSC-CM typically lacked a prominent phase I repolarization notch, which was probably linked to the near absence of I_to,f_ currents (Fig. 5C and D). In contrast, cells transduced with the cTnT-Kv4.3/KChIP2.1 construct (72 h post transduction) developed a robust I_to,f_ whose magnitudes and inactivation kinetics were similar to human I_to,f_, as seen in the other cell types examined here (Fig. 5C and supplementary Fig. S3A). As a result, the evoked APs of the transduced cells appeared to have a more mature ventricular phenotype characterized by a prominent phase I notch, lower early plateau membrane potential and shorter APD90 (Fig. 5D and supplementary Fig. S3B; data summarized in supplementary Table S2).

**Fig. 6:**
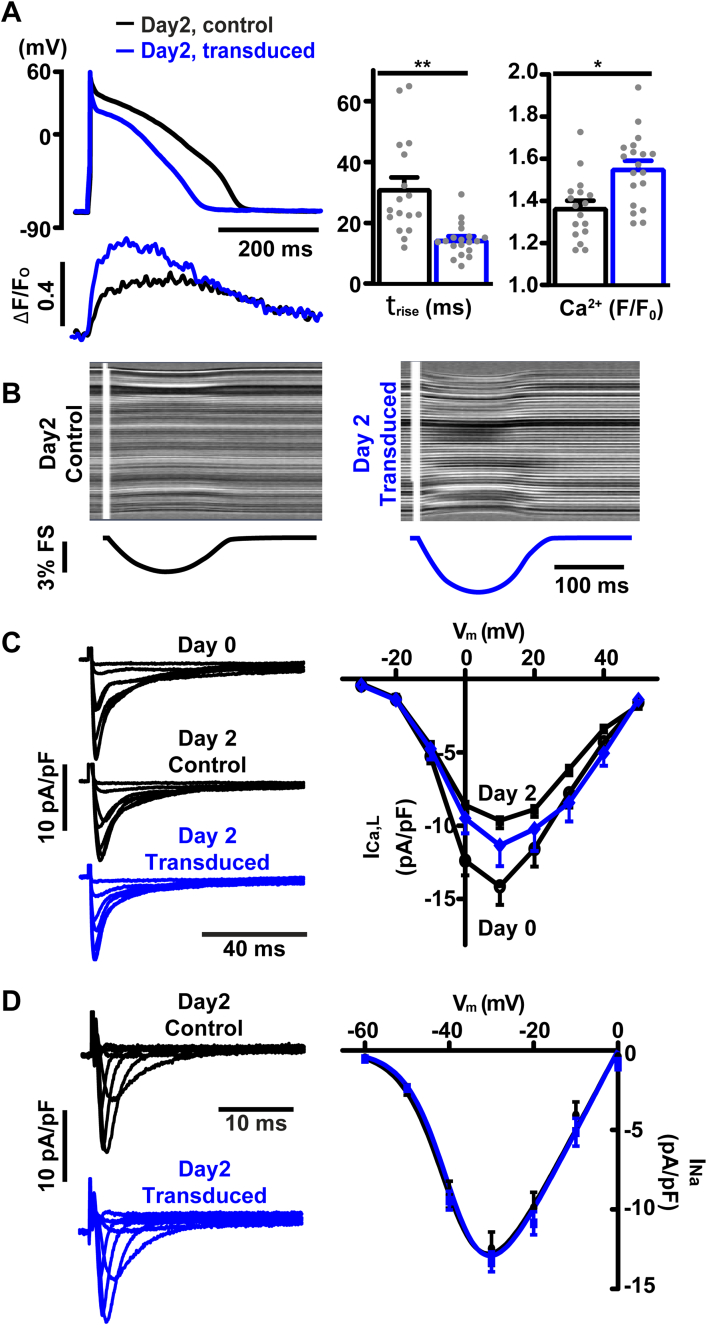
Effect of bicistronic construct expression on ECC, I_Ca,L_ and I_Na_ currents. A. Exemplar APs from transduced (blue) and control (black) cultured myocytes. These cells expressed moderate levels of I_to,f_ (cf. Fig. 5A). The lower panel shows corresponding Ca^2+^ transients at a cycle length of 1 s. Changes in the rate of rise (untransduced 31.1 ± 3.9, *n* = 17 vs transduced 14.4 ± 1.2, n/N 19/7; *p* = 0.002 nested t-test) and amplitude of the Ca^2+^ transient are summarized on the right (untransduced 1.37 ± 0.04 vs transduced 1.55 ± 0.045; *p* = 0.02, nested t-test n/N 19/7). ** *p* < 0.01; * *p* < 0.05. B. Contraction measured by recording transmitted light line scan images of the cells. The stripes reflect sarcomeric structures. The change in distance between the ends of the cell give the % fractional shortening (FS) as shown in the lower panels (data summarized in Table 1). C. I_Ca,L_ evoked by pulses from −30 mV to +30 mV in acutely isolated myocytes (day 0) and after 40 h in culture, with and without viral transduction (data summarized in Table 1). D. Exemplar I_Na_ recordings with 5 mmol/l [Na^+^] (from a holding potential of −120 mV to −60 mV in 10 mV increments to 0 mV) are shown on the left. The current-voltage relationship for I_Na_ is shown at right; there was no significant change in either amplitude or half maximal activation voltage (V_0.5,_). I_Na_ density at −30 mV: −12.5 ± 1.1 pA/pF (n/*N* = 12/2) in untransduced vs −13.3 ± 0.6 pA/pF (n/*N* = 11/2) in transduced myocytes (*p* = 0.52, nested t-test). V_0.5_: −39.0 ± 0.5 mV in untransduced vs −38.4 ± 0.6 mV in transduced myocytes (*p* = 0.45, unpaired t-test). Error bars show mean ± s.e.m. (For interpretation of the references to colour in this figure legend, the reader is referred to the web version of this article.)

Fig. 6A illustrates the effect of using the Kv4.3/KChIP2.1 vector to introduce a moderate phase 1 repolarization, similar to that seen in humans, on Ca^2+^ handling in rabbit epicardial myocytes. The significant reduction in APD_20_ due to Kv4.3/KChIP2.1 transduction was associated with an ~2-fold increase in the rate of rise and a smaller increase in the peak amplitude of the Ca^2+^ transient (Fig. 6A right). Since APD_90_ was reduced in these exeriments by Kv4.3/KChIP2.1 expression (Table 1), this improvement in ECC was not due to the effect of AP duration on ECC [38]. The changes in Ca^2+^ transient amplitude and time course due to Kv4.3/KChIP2.1 transduction increased contraction amplitude as shown in Fig. 6B.

Fig. 6C shows that the change in Ca^2+^ transient amplitude was not due to a change in I_Ca,L_ density (Table 1), although the I_Ca,L_ density after culture was slightly reduced when compared to freshly isolated cells (see Table 1 and [39]). Control experiments showed that the small increase in I_Ca,L_ after 2 days in culture between control and transduced cells seen in Fig. 6C could not be explained by adenoviral exposure per se (Fig. S2C). Similarly, bicistronic transgene expression did not affect the amplitude or voltage-dependence of activation of I_Na_ (Fig. 6D) nor the resting membrane potential and peak AP amplitude (Fig. S2E). Collectively, these data suggest that modifying phase 1 repolarization by introducing moderate I_to,f_ current can improve ECC without affecting biophysical properties of other major ionic currents involved in ECC.

## 4 Discussion

The results of this study show that a single P2A-linked bicistronic construct could deliver simultaneous expression of both the Kv4.3 pore-forming α-subunits and auxiliary KChIP2 β-subunits in cardiomyocytes, which are two key components of a physiological I_to,f_. While K^+^ ion channel expression in mouse models has been used extensively to study channel function and the consequences of disease mutations [3], our goal here was to modify a high plateau AP that has a small or absent phase 1 notch, similar to failing human cells. By making a robust phase 1 repolarization from the key components of human I_to,f_ in a single viral construct we could then examine how such a gene transfer might affect AP morphology and ECC. When expressed in rabbit ventricular myocytes (where the endogenous I_to,f_ is comparatively small, see Fig. 4A and [21]), the bicistronic transgenes produced an I_to,f_ very similar to that reported for human ventricular cardiomyocytes with slowed inactivation at positive V_m_ and rapid recovery from inactivation. Data from hiPSC-CMs showed that, under the control of a cardiac specific promotor, the Kv4.3 /KChIP2.1 construct could produce an ~10 pA/pF I_to,f_. As was seen in rabbit cardiac myocytes, this current produced a clear phase 1 in the AP. Unfortunately, our hiPSC-CMs did not develop a mature ventricular cardiac myocyte morphology with TT and sarcomeres in register (not shown), precluding an examination into the effects on ECC. However, our experiments on rabbit myocytes clearly show that manipulating magnitude of I_to,f_ by stoichiometric expression of Kv4.3 and KChIP2.1 can not only shape the trajectory of phase I repolarization but also reduce AP triangulation, APD variability and enhance ECC. This suggests that, with appropriate bicistronic expression of Kv4.3 and KChIP2 with a suitable cardiac promotor, we might be able to ameliorate the loss of I_to,f_ seen in failing human hearts to improve ECC without promoting arrhythmogenesis.

### 4.1 Kv4.3/KChIP2.1 complex stoichiometry and interactions

The employment of bicistronic approaches not only overcomes the need for two vectors to deliver essential α- and β-subunits of an ion channel, but should also provide near equimolar expression of the two proteins [40]. Our electrophysiological data from HEK293 cells showed that co-expression of Kv4.3 and KChIP2.1 with the bicistronic vector slows the inactivation kinetics and accelerates the recovery of Kv4.3-encoded currents, consistent with previous work examining co-assembly of Kv4.3 with KChIP2 [25,41]. It was notable that no further change of I_to,f_ was detected in cells expressing excess KChIP2.1, indicating that stoichiometric expression of KChIP2.1 was achieved and was sufficient to produce the full effect of KChIP2.1. This result is consistent with the observation that the biophysical properties of Kv4.3 currents change with increasing KChIP2.1 expression until the molar ratio reaches 1:1 [32]. These findings also concur with crystallographic data suggesting that a single KChIP1 or KChIP4 molecule (both of which share high sequence homology with KChIP2.1) binds to adjacent α-subunits of a Kv4 tetrameric channel resulting in a 4:4 Kv4:KChIP multimer [42,43] and this stoichiometry is also suggested by electron microscopy [44].

KChIP2 and/or Kv4.3 expression has been reported to alter I_Ca,L_ and I_Na_ as well as other regulatory systems [[45], [46], [47], [48]]. Despite these concerns, our electrophysiological data suggests that the current density-voltage relationships for I_Ca,L_ and I_Na_ were not altered, in accord with the effect of expressing Kv4.3 alone in guinea pig myocytes [49]. The apparent lack of effect of exogenous KChIP2 expression on I_Ca,L_ and I_Na_ in our experiments may be due to the the fact that we are co-expressing stoichiometric Kv4.3: If the Kv4.3 channel binds KChIP2.1 with high affinity (relative to the expression level), most of the exogenous KChIP2.1 protein would be bound to the expressed Kv4.3 and so the availability of free KChIP2.1 would not markedly increase.

### 4.2 Subcellular distribution of Kv4.3 and KChIP2.1

KChIPs have been shown to migrate to the surface membrane in the presence of A-type potassium channels [50]. We observed a strong overlap of fluorescently tagged Kv4.3 and KChIP2.1 with a high Pearson's correlation coefficient in both transfected HEK293 cells and transduced cardiomyocytes. Most of the Kv4.3/KChIP2.1 labelling was seen in Cav3 labelled TT and peripheral sarcolemma regions (Fig. 3B), and visually, the distribution of the expressed Kv4.3/KChIP2.1 appeared similar to images of these I_to,f_ components in human cardiomyocytes [41]. Earlier patch-clamp recordings of I_to_ from control and detubulated rat sub-epicardial myocytes also suggested that I_to_ currents are uniformly distributed between the surface membrane and the TT regions [51] in broad agreement with our imaging results. That the expressed proteins appear near the cell surface is not unexpected given the electrophysiological data, but the concentration of these proteins near TTs is an important indicator that normal trafficking is occurring. In principle, correct trafficking should help limit (possible) ER stress that may occur if a strong promotor (e.g. CMV) causes abnormal accumulation of proteins in the ER [52].

### 4.3 I_to,f_ transduction alters AP morphology and Ca^2+^ handling

Virally transduced rabbit LV cardiomyocytes produced a current which was kinetically similar to that reported for non-failing human ventricular I_to,f_ [30] but quite distinct from the smaller intrinsic rabbit I_to_. Pharmacologically, the augmented I_to,f_ recorded from transduced cardiomyocytes was also similar to that reported for native channels. It was both effectively blocked by 4-AP at a similar concentrations to that reported for native human channels [9] and 5 μmol/l NS5806 (which is not an agonist without KChIP2 expression [29]), increased the expressed I_to,f_ by 32% in good agreement with the results of a previous study [32].

Changes in the level of transgene expression allowed us to experimentally examine the effect of different I_to,f_ densities in rabbit LV cardiomyocytes. It is notable that as the measured I_to,f_ density increased, the AP morphology changed from rabbit toward human, then dog and finally rodent phenotypes. That this occurred at I_to,f_ densities that are in reasonable agreement with reported current densities for these species strongly suggests that I_to,f_ is an important current component to program later AP trajectory [[53], [54], [55]], although there are other (well-known) species differences in repolarizing K^+^ channel currents (e.g. [56]) that affect later AP timecourse that will not be discussed here. Expression of Kv4.3 in guinea pig myocytes depressed the AP plateau in direct proportion to the expressed current density and shortened APD90 [49]. In the present study, neither the plateau (as shown by the duration of APD20) nor APD90 was linearly related to I_to,f_ current density (Fig. 5B) and this may be explained by the bicistronic I_to,f_ having more complete inactivation during the AP due to the co-expression of KChIP2.1 (Fig. S2). The sudden change in AP morphology at ~12 pA/pF (at +40 mV) suggests that this level of expression might be a therapeutic limit to the degree that I_to,f_ should be augmented in vivo. This observation was a part of our motivation for testing the effect of a different promotor (cTnT) in hiPSC-CMs and, under control of this promotor, we were able to produce an I_to,f_ of about this level (Fig. 5C).

Computer simulations have also shown an abrupt reduction in AP duration at ~0.13 nS/pF (corresponding to ~14 pA/pF) [57] in agreement with our results. While such an AP collapse might recapitulate Brugada syndrome [17], our data shows that controlled Kv4.3/KChIP2.1 expression and restoration of I_to,f_ could be beneficial in reducing AP triangulation and beat-to-beat variability (the latter probably reflecting a more synchronous Ca^2+^ release [16,58]). That modulating these key components of I_to,f_ might reduce the risk of arrhythmia is also supported by the observation that increasing KChIP2 expression (or inhibiting miR-34) under pathologic conditions prevented reentry arrhythmias [46].

Moderate augmentation of phase I repolarization by the expression of bicistronic transgenes produced an improvement in the initial phase of the Ca^2+^ transient. Our previous study examining Ca^2+^ transients evoked by human AP waveforms suggested that synchronous SR Ca^2+^ release was compromised when failing human AP loses the phase 1 notch [14]. Modest phase 1 hyperpolarization from positive potentials can increase the driving force for trigger I_Ca,L_ [14] and thereby synchronize and optimise SR Ca^2+^ release - provided phase 1 occurs over the positive limb of the I_Ca,L_ current-voltage relation (i.e. at membrane potentials greater than ~ + 10 mV -see Fig. 6C). However in dogs, the intrinsic phase 1 tends to be deeper, moving I_Ca,L_ onto its activating limb where increasing phase 1 repolarization will decrease I_Ca,L_ [59]. We suggest this difference may help explain the reported failure of the I_to_ agonist NS5806 to augment contractility in dog models [19,60].

## 5 Conclusions and limitations for translation

We have shown that a robust I_to,f_, which requires both Kv4.3 and KChIP2.1, can be produced in cardiomyocytes by using a bicistronic gene construct and viral transduction. It follows that therapy for other loss of function channelopathies, either from defects in the channel itself and/or accessory subunits, may be also be amenable to bicistronic viral transduction. An important aspect of this approach is the control of the stoichiometry between the exogenous gene products and hence relative protein expression levels. We suggest that, in principle, the loss of I_to,f_ and phase 1 repolarization (and AP prolongation) in human heart failure may be correctable by similar methods and this may confer an improvement in ECC and reduction in arrhythmia risk.

On the other hand, coupling the bicistronic construct to a very strong promotor can initiate AP collapse, although whether this would occur in intact hearts is far from clear. This uncertainty arises from the non-uniform nature of viral transduction and transgene expression that will reduce the average Kv4.3/KChIP protein expression level to less than the maximum possible in individual cells. Electrical coupling between cells will then average the local I_to,f_ density over the electrical space constant of the tissue to limit transduction effects on the AP. Similarly, it is unclear whether regional differences in native I_to,f_ would be completely removed by bicistronic gene transduction. Thus, while we show that a cTnT promotor can produce an I_to,f_ in hiPSC-CM cells that is of similar magnitude to that seen in normal human ventricular cells, it is possible that some other (as yet unidentified) promotor or the addition of a suitable miRNA may, in the future, enable the clinician to properly recapitulate the normal regional differences in I_to,f_ (and other currents) in diseased hearts.

## Funding

This work was supported by a Medical Research Council U.K. program grant (MR/N002903/1).

## Author contributions

Conceptual design MBC; Experimental design NW, JCH, MBC. NW, EDF, and SCH carried out experiments. JCH and MBC secured funding. All authors contributed to manuscript preparation.

## Data availability

The data underlying this article will be shared on reasonable request to the corresponding author.

## Declaration of competing interest

None declared.

## Appendix A Supplementary data

Supplementary material Image 1

## References

[1] Oudit G. (2001). The molecular physiology of the cardiac transient outward potassium current (Ito) in normal and diseased myocardium. J. Mol. Cell. Cardiol..

[2] Rosati B., Pan Z., Lypen S., Wang H.-S., Cohen I., Dixon J.E. (2001). Regulation of KChIP2 potassium channel beta subunit gene expression underlies the gradient of transient outward current in canine and human ventricle. J. Physiol. Lond..

[3] Nerbonne J.M., Nichols C.G., Schwarz T.L., Escande D. (2001). Genetic manipulation of cardiac K(+) channel function in mice: what have we learned, and where do we go from here?. Circ. Res..

[4] Näbauer M., Beuckelmann D.J., Erdmann E. (1993). Characteristics of transient outward current in human ventricular myocytes from patients with terminal heart-failure. Circ. Res..

[5] Nerbonne J.M., Kass R.S. (2005). Molecular physiology of cardiac repolarization. Physiol. Rev..

[6] Patel S.P., Campbell D.L. (2005). Transient outward potassium current, “Ito,” phenotypes in the mammalian left ventricle: underlying molecular, cellular and biophysical mechanisms. J. Physiol. Lond..

[7] Zygmunt A.C., Gibbons W.R. (1991). Calcium-activated chloride current in rabbit ventricular myocytes. Circ. Res..

[8] Köster O.F., Szigeti G.P., Beuckelmann D.J. (1999). Characterization of a [Ca2+]i-dependent current in human atrial and ventricular cardiomyocytes in the absence of Na+ and K. Cardiovasc. Res..

[9] Näbauer M., Beuckelmann D.J., Uberfuhr P., Steinbeck G. (1996). Regional differences in current density and rate-dependent properties of the transient outward current in subepicardial and subendocardial myocytes of human left ventricle. Circulation..

[10] Radicke S., Cotella D., Graf E., Banse U., Jost N., Varro A. (2006). Functional modulation of the transient outward current Ito by KCNE β-subunits and regional distribution in human non-failing and failing hearts. Cardiovasc. Res..

[11] Soltysinska E., Olesen S.-P., Christ T., Wettwer E., Varró A., Grunnet M. (2009). Transmural expression of ion channels and transporters in human nondiseased and end-stage failing hearts. Pflugers Arch..

[12] Sah R., Ramirez R.J., Backx P.H. (2002). Modulation of Ca2+ release in cardiac myocytes by changes in repolarization rate. Circ. Res..

[13] Harris D.M., Mills G.D., Chen X., Kubo H., Berretta R.M., Votaw V.S. (2005). Alterations in early action potential repolarization causes localized failure of sarcoplasmic reticulum Ca2+ release. Circ. Res..

[14] Cooper P.J., Soeller C., Cannell M.B. (2010). Excitation-contraction coupling in human heart failure examined by action potential clamp in rat cardiac myocytes. J. Mol. Cell. Cardiol..

[15] Smith G. (2007). Matters of the heart: the physiology of cardiac function and failure. Exp. Physiol..

[16] Fowler E.D., Wang N., Hezzell M., Chanoit G., Hancox J.C., Cannell M.B. (2020). Arrhythmogenic late Ca2+ sparks in failing heart cells and their control by action potential configuration. Proc. Natl. Acad. Sci..

[17] Calloe K., Cordeiro J.M., Di Diego J.M., Hansen R.S., Grunnet M., Olesen S.-P. (2009). A transient outward potassium current activator recapitulates the electrocardiographic manifestations of Brugada syndrome. Cardiovasc. Res..

[18] Wang S., Rodríguez-Mañero M., Ibarra-Cortez S.H., Kreidieh B., Valderrábano L., Hemam M. (2019). NS5806 induces electromechanically discordant alternans and arrhythmogenic voltage-calcium dynamics in the isolated intact rabbit heart. Front. Physiol..

[19] Dong M., Yan S., Chen Y., Niklewski P.J., Sun X., Chenault K. (2010). Role of the transient outward current in regulating mechanical properties of canine ventricular myocytes. J. Cardiovasc. Electrophysiol..

[20] Calloe K., Nof E., Jespersen T., Di Diego J.M., Chlus N., Olesen S.-P. (2011). Comparison of the effects of a transient outward potassium channel activator on currents recorded from atrial and ventricular cardiomyocytes. J. Cardiovasc. Electrophysiol..

[21] Cheng H., Cannell M.B., Hancox J.C. (2017). Differential responses of rabbit ventricular and atrial transient outward current (Ito) to the Ito modulator NS5806. Phys. Rep..

[22] Decher N., Uyguner O., Scherer C.R., Karaman B., Yüksel-Apak M., Busch A.E. (2001). hKChIP2 is a functional modifier of hKv4.3 potassium channels: cloning and expression of a short hKChIP2 splice variant. Cardiovasc. Res..

[23] Lainez S., Doray A., Hancox J.C., Cannell M.B. (2018). Regulation of Kv4.3 and hERG potassium channels by KChIP2 isoforms and DPP6 and response to the dual K+channel activator NS3623. Biochem. Pharmacol..

[24] Dilks D., Ling H.P., Cockett M., Sokol P., Numann R. (1999). Cloning and expression of the human kv4.3 potassium channel. J. Neurophysiol..

[25] Abbott G.W. (2017). β subunits functionally differentiate human Kv4.3 potassium channel splice variants. Front. Physiol..

[26] Kim J.H., Lee S.-R., Li L.-H., Park H.-J., Park J.-H., Lee K.Y. (2011). High cleavage efficiency of a 2A peptide derived from porcine teschovirus-1 in human cell lines, zebrafish and mice. PLoS One.

[27] Akar F.G. (2004). Phenotypic differences in transient outward K+ current of human and canine ventricular myocytes: insights into molecular composition of ventricular Ito. Am. J. Physiol. Heart Circ. Physiol..

[28] Takimoto K., Yang E.-K., Conforti L. (2002). Palmitoylation of KChIP splicing variants is required for efficient cell surface expression of Kv4.3 channels. J. Biol. Chem..

[29] Lundby A., Jespersen T., Schmitt N., Grunnet M., Olesen S.P., Cordeiro J.M. (2010). Effect of the Ito activator NS5806 on cloned Kv4 channels depends on the accessory protein KChIP2. Brit. J. Pharmacol..

[30] Johnson E.K., Springer S.J., Wang W., Dranoff E.J., Zhang Y., Kanter E.M. (2018). Differential expression and remodeling of transient outward potassium currents in human left ventricles. Circ. Arrhythm. Electrophysiol..

[31] Kehl S.J. (2017). A model of the block of voltage-gated potassium Kv4.2 ionic currents by 4-Aminopyridine. J. Pharmacol. Exp. Ther..

[32] Calloe K., Soltysinska E., Jespersen T., Lundby A., Antzelevitch C., Olesen S.-P. (2010). Differential effects of the transient outward K(+) current activator NS5806 in the canine left ventricle. J. Mol. Cell. Cardiol..

[33] Zhang H., Zhang H., Wang C., Wang Y., Zou R., Shi C. (2020). Auxiliary subunits control biophysical properties and response to compound NS5806 of the Kv4 potassium channel complex. FASEB J..

[34] Jost N., Virág L., Comtois P., Ordög B., Szuts V., Seprényi G. (2013). Ionic mechanisms limiting cardiac repolarization reserve in humans compared to dogs. J. Physiol. Lond..

[35] Shimoni Y., Severson D., Giles W. (1995). Thyroid status and diabetes modulate regional differences in potassium currents in rat ventricle. J. Physiol. Lond..

[36] Hondeghem L.M., Carlsson L., Duker G. (2001). Instability and triangulation of the action potential predict serious proarrhythmia, but action potential duration prolongation is antiarrhythmic. Circulation..

[37] Pueyo E., Dangerfield C.E., Britton O.J., Virág L., Kistamás K., Szentandrassy N. (2016). Experimentally-based computational investigation into beat-to-beat variability in ventricular repolarization and its response to ionic current inhibition. PLoS One.

[38] Bouchard R.A., Clark R.B., Giles W.R. (1995). Effects of action potential duration on excitation-contraction coupling in rat ventricular myocytes. Action potential voltage-clamp measurements. Circ. Res..

[39] Mitcheson J.S., Hancox J.C., Levi A.J. (1996). Action potentials, ion channel currents and transverse tubule density in adult rabbit ventricular myocytes maintained for 6 days in cell culture. Pflugers Arch..

[40] Szymczak A.L., Workman C.J., Wang Y., Vignali K.M., Dilioglou S., Vanin E.F. (2004). Correction of multi-gene deficiency in vivo using a single “self-cleaving” 2A peptide-based retroviral vector. Nat. Biotechnol..

[41] Deschenes I. (2002). Regulation of Kv4.3 current by KChIP2 splice variants: a component of native cardiac Ito?. Circulation..

[42] Pioletti M., Findeisen F., Hura G.L., Minor D.L. (2006). Three-dimensional structure of the KChIP1-Kv4.3 T1 complex reveals a cross-shaped octamer. Nat. Struct. Mol. Biol..

[43] Wang K. (2008). Modulation by clamping: Kv4 and KChIP interactions. Neurochem. Res..

[44] Kim L.A., Furst J., Gutierrez D., Butler M.H., Xu S.H., Goldstein S. (2004). Three-dimensional structure of I-to: Kv4.2-KChIP2 ion channels by electron microscopy at 21 angstrom resolution. Neuron..

[45] Thomsen M.B., Wang C., Ozgen N., Wang H.-G., Rosen M.R., Pitt G.S. (2009). Accessory subunit KChIP2 modulates the cardiac L-type calcium current. Circ. Res..

[46] Nassal D.M., Wan X., Liu H., Maleski D., Ramirez-Navarro A., Moravec C.S. (2017). KChIP2 is a core transcriptional regulator of cardiac excitability. Elife..

[47] Keskanokwong T., Lim H.J., Zhang P., Cheng J., Xu L., Lai D. (2011). Dynamic Kv4.3-CaMKII unit in heart: an intrinsic negative regulator for CaMKII activation. Eur. Heart J..

[48] Portero V., Wilders R., Casini S., Charpentier F., Verkerk A.O., Remme C.A. (2018). KV4.3 expression modulates NaV1.5 sodium current. Front. Physiol..

[49] Hoppe U.C., Marbán E., Johns D.C. (2000). Molecular dissection of cardiac repolarization by in vivo Kv4.3 gene transfer. J. Clin. Invest..

[50] An W.F., Bowlby M.R., Betty M., Cao J., Ling H.-P., Mendoza G. (2000). Modulation of A-type potassium channels by a family of calcium sensors. Nature..

[51] Komukai K., Brette F., Yamanushi T.T., Orchard C.H. (2002). K(+) current distribution in rat sub-epicardial ventricular myocytes. Pflugers Arch..

[52] Xu C., Bailly-Maitre B., Reed J.C. (2005). Endoplasmic reticulum stress: cell life and death decisions. J. Clin. Invest..

[53] Dong M., Sun X., Prinz A.A., Wang H.S. (2006). Effect of simulated I(to) on guinea pig and canine ventricular action potential morphology. Am. J. Physiol. Heart Circ. Physiol..

[54] Hund T.J., Rudy Y. (2004). Rate dependence and regulation of action potential and calcium transient in a canine cardiac ventricular cell model. Circulation..

[55] Sala L., Hegyi B., Bartolucci C., Altomare C., Rocchetti M., Váczi K. (2018). Action potential contour contributes to species differences in repolarization response to β-adrenergic stimulation. Europace..

[56] Zicha S., Moss I., Allen B., Varró A., Papp J., Dumaine R. (2003). Molecular basis of species-specific expression of repolarizing K+ currents in the heart. Am. J. Physiol. Heart Circ. Physiol..

[57] Greenstein J.L., Wu R., Po S., Tomaselli G.F., Winslow R.L. (2000). Role of the calcium-independent transient outward current I(to1) in shaping action potential morphology and duration. Circ. Res..

[58] Cordeiro J.M., Malone J.E., Di Diego J.M., Scornik F.S., Aistrup G.L., Antzelevitch C. (2007). Cellular and subcellular alternans in the canine left ventricle. Am. J. Physiol. Heart Circ. Physiol..

[59] Sah R., Ramirez R.J., Oudit G.Y., Gidrewicz D., Trivieri M.G., Zobel C. (2002). Regulation of cardiac excitation-contraction coupling by action potential repolarization: role of the transient outward potassium current (Ito). J. Physiol. Lond..

[60] Maleckar M.M., Lines G.T., Koivumäki J.T., Cordeiro J.M., Calloe K. (2014). NS5806 partially restores action potential duration but fails to ameliorate calcium transient dysfunction in a computational model of canine heart failure. Europace..

[61] Rath A., Glibowicka M., Nadeau V.G., Chen G., Deber C.M. (2009). Detergent binding explains anomalous SDS-PAGE migration of membrane proteins. Proc. Natl. Acad. Sci..

