# Supplementary material for: Inducing Ito,f and phase 1 repolarization of the cardiac action potential with a Kv4.3/KChIP2.1 bicistronic transgene
Source: J Mol Cell Cardiol. 2022 Mar;164:29–41. doi: 10.1016/j.yjmcc.2021.11.004 (PMC8884339; doi:10.1016/j.yjmcc.2021.11.004)
Supplement: Supplementary file 1 — Supplementary material [file mmc1.pdf]

## Supplemental Materials

Supplementary Figure S1; S2; S3

Supplementary Table S1; S2

Expanded Materials & Methods

Supplementary References

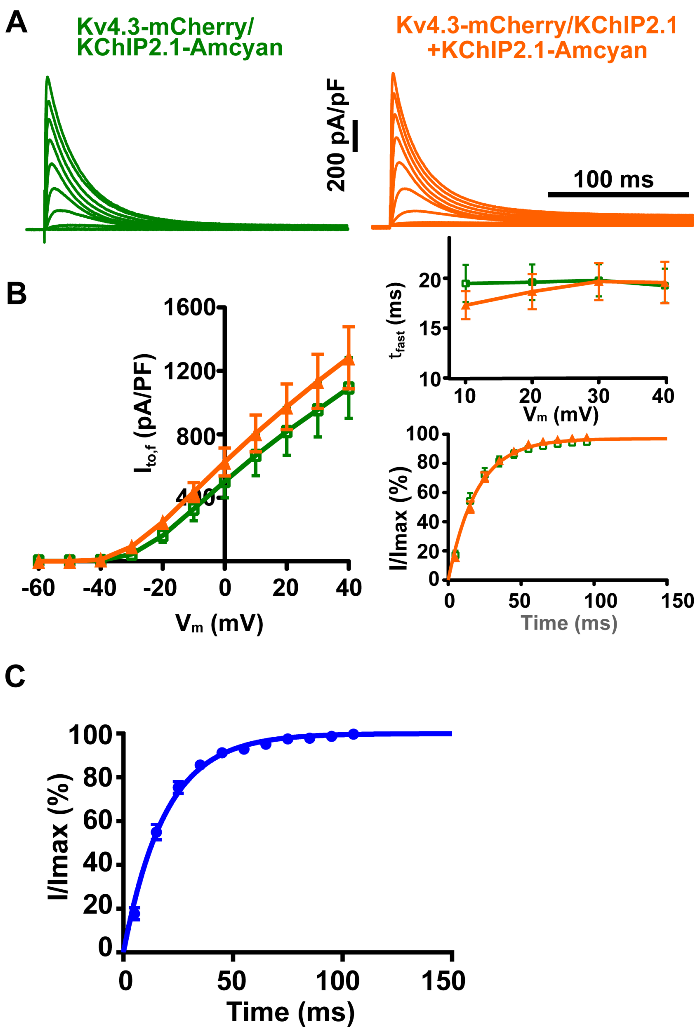


**Supplementary Figure S1. A**. Currents recorded from HEK293 cells co-transfected with both the bicistronic vector Kv4.3-mCherry-P2A-KChIP2.1 (green) and additional KChIP2.1-Amcyan at a plasmid ratio of 1:1 (orange). Cells expressing additional KChIP2.1 were selected on the basis of exhibiting both mCherry and Amcyan labelling. Summary data and number of replicates for the different groups are given in Supplementary Table S1. **B.** Voltage dependence of activation, the kinetics of current inactivation and time course of recovery from inactivation were not changed by additional KChIP2.1 expression. Data colored as in panel **A. C.** Time course of recovery of the expressed $I_{to,f}$ in cardiac myocytes (n = 10; N = 3 ). To examine the kinetics of the $I_{to,f}$ recovery from inactivation, myocytes were held at -70 ms for various interpulse intervals ranging from 5 to 105 ms. Error bars show mean ± s.e.m.

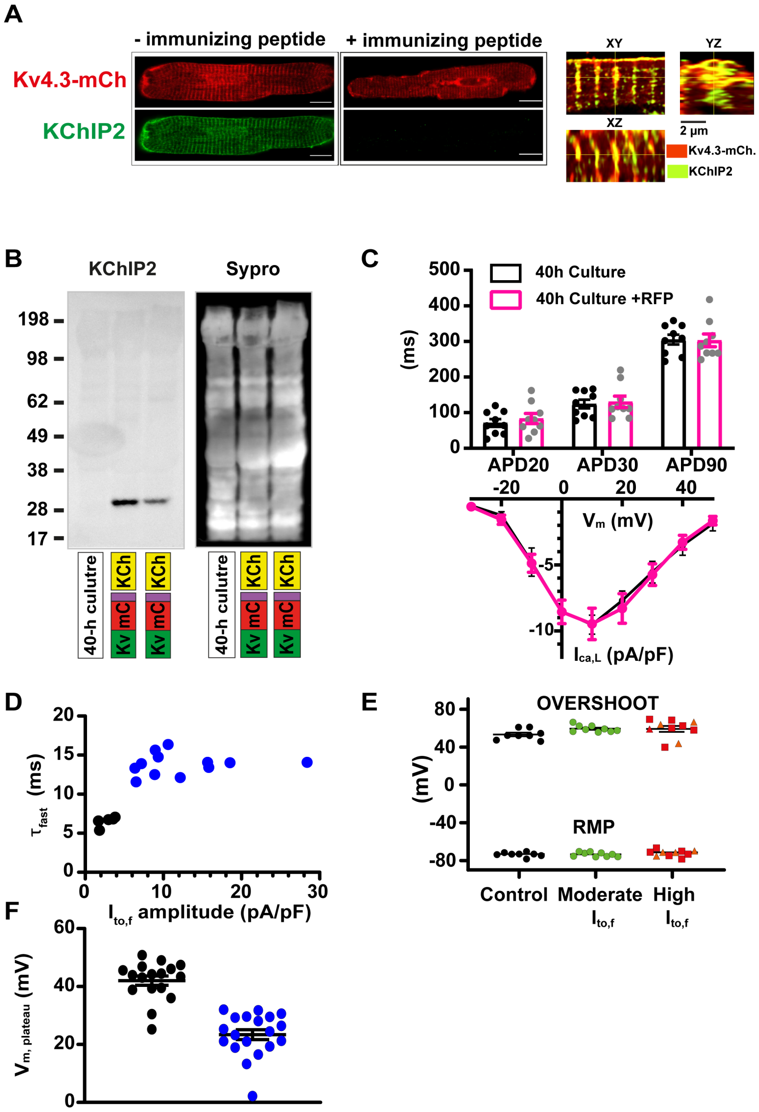


**Supplementary Figure S2**. **A.** Specificity of anti-KChIP2 antibody in immunolabelling of transduced ventricular cardiomyocytes. In the representative confocal images, the cardiomyocytes were transduced with the bicstronic Kv4.3/KChIP2.1 construct, as shown by the mCherry fluorescence. Note that preincubation with KChIP2 immunizing peptide blocked all labelling showing the specificity of the primary and secondary antibodies. Scale bar: 10 µm. The right-hand panel shows confocal orthogonal sections at the level of a t-tubule with Kv4.3 in red overlaid with KChIP2.1 in green. The strong yellow color suggests colocalization at the surface membrane and along t-tubules. **B.** Western blot analysis of KChIP2 expression in untransduced and transduced cardiomyocytes. A strong KChIP2.1 band at a molecular weight of ~30 kDa corresponding to the KChIP2.1 isoform was detected in myocytes transduced with Kv4.3-mCherry-P2A-KChIP2.1. Higher molecular weight bands between 98 and 198 KDa were not found in transduced myocyte lysates, suggesting a complete cleavage of the bicistronic construct into separate Kv4.3 and KChIP2.1 proteins. Two independent replicates of transduced myocyte samples are shown. **C**. APD morphologies and $I_{ca,L}$ densities were not different

in myocytes transduced with a control mApple-based red fluorescent protein (RFP in magenta) compared with untransduced myocytes in culture (black). APD20: 70.9 ±10.8 ms in untransduced *vs* 83.6 ± 14.1 in control transduced cells, P =0.57; APD30: 124.5 ±12.1 ms in untransduced *vs* 130.5 ± 16.0 in control transduced cells, P =0.88; APD90: 305.2 ±13.9 ms in untransduced *vs* 303.0 ± 18.0 in control transduced cells, P =0.92 (n/N = 9/3). $I_{ca,L}$ at +10 mV: -9.5 ±0.7 pA/pF in untransduced *vs* -9.5 ±1.2 pA/PF in control transduced cells, P =0.97 (N/n= 8/3 all P-values from nested t-tests). **D.** The fast inactivating component of the exogenous $I_{to,f}$ (blue circle) in transduced myocytes was markedly slower than that of the intrinsic $I_{to,f}$ in cultured, untransduced cells (black circle), but its kinetics did not change with increasing amplitude of $I_{to,f}$ current density (measured at +40 mV; $R^2$= 0.015). **E.** Increasing density of $I_{to,f}$ as shown in Fig. 5B did not alter resting membrane potential (RMP) (P = 0.22, one way ANOVA) or AP overshoot (P = 0.16, one way ANOVA) of the transduced myocytes. **F**. AP plateau potentials of the transduced and control (untransduced) myocytes presented in Fig. 6A (measured at 20 ms after the initiation of the action potential upstroke). Untransduced 42.0 ± 1.6 mV, n=17 *vs* transduced 23.4 ± 1.7 mV, n/N 19/7; p < 0.0001 from nested t-test. Error bars show mean ± s.e.m.

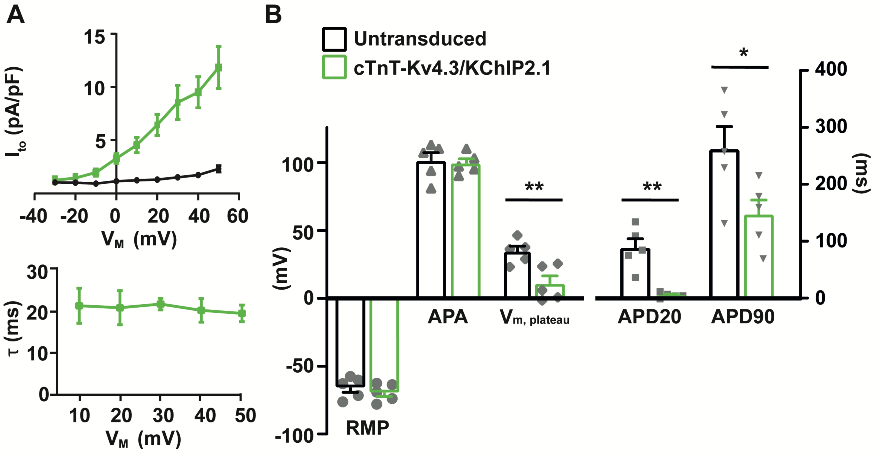


**Supplementary Figure S3**. **A**. Mean I-V relations of $I_{to,f}$ recorded in untransduced iPSC-CMs (black) and iPSC-CMs expressing bicistronic Kv4.3-mCherry/KChIP2.1 (green) under the cardiac-specific cTnT promoter (upper panel). Lower panel shows the time constants of the fast inactivating components of the exogenous $I_{to,f}$ plotted as a function of membrane potential. **B**. Effects of cTnT-Kv4.3/KChIP2.1 transgenes on AP parameters. RMP:

resting membrane potential. $V_{m,\ plateau}$: Membrane potential of the early plateau phase. APA: AP amplitude. APD* p<0.05; ** p<0.01 by Student's t-test. Error bars show mean ± s.e.m.

**Supplementary Table S1**. Properties of $I_{to,f}$ mediated by transgene expression in HEK293 cells. Values are mean ± s.e.m.

| | **Kv4.3-mCherry /KChIP2.1** | **Kv4.3-mCherry /KChIP2.1-Amcyan** | **Kv4.3/KChIP2.1 +KChIP2.1-Amcyan** |
|---|---|---|---|
| $I_{to,f}$ amplitude @ +40 mV (pA/pF) | 1164 ± 167 (n = 19) | 1092 ± 193 (n = 10) | 1284 ± 196 (n = 12) |
| Decay $\tau_{fast}$ @ +40 mV (ms) | 21.2 ± 1.6 (n = 18) | 19.4 ± 1.7 (n = 10) | 19.6 ± 2.1 (n = 8) |
| Decay $\tau_{slow}$ @ +40 mV (ms) | 53.5 ± 4.7 (n = 18) | 51.5 ± 3.8 (n = 10) | 58.1 ± 7.2 (n = 8) |
| Recovery time constant @ -80 mV (ms) | 20.4 ± 0.7 (n = 15) | 19.5 ± 0.9 (n = 10) | 19.7 ± 0.6 (n = 7) |

**Supplementary Table S2**. Summary of $I_{to}$ current densities and AP profiles measured in control (CN) iPSC-CMs and iPSC-CMs transduced with cTnT-Kv4.3/KChIP2.1 construct. (N=5 for both goups; * p<0.05; ** p<0.01; *** p <0.001). Values are mean ± s.e.m and P-values from unpaired t-tests.

| | **Control (CN)** | **cTnT-Kv4.3/KChIP2.1** | **p-value** |
|---|---|---|---|
| Cell capacitance (pF) | 32.8 ± 4.7 | 35.9 ± 5.2 | 0.666 |
| $I_{to}$ peak @ +40 mV (pA/pF) | 1.5 ± 0.2 | 9.5 ± 1.7*** | <0.001 |
| RMP | -65.7 ± 3.5 | -69.4 ± 3.0 | 0.442 |
| APA | 101.2 ± 6.0 | 99.3 ± 3.4 | 0.790 |
| $V_{m,\ plateau}$ (mV) | 34.5 ± 4.0 | 10.8 ± 5.7 ** | 0.009 |
| APD20 | 91.6 ± 16.5 | 8.7 ± 2.2 ** | 0.0011 |
| APD50 | 159.5 ± 28.9 | 61.3 ± 14.8 * | 0.016 |

| | | | |
|---|---|---|---|
| APD90 | 265.4 ± 40.4 | 150.5 ± 25.8 * | 0.043 |

**Supplementary Materials & Methods**

***Cell culture, transfection and electrical recording***

Human embryonic kidney cells (HEK293; European Collection of Cell Cultures, Porton Down, UK) were cultured in Dulbecco's modified Eagle's medium (ThermoFisher Scientific, Life Technologies division, Paisley, UK) supplemented with 5% (v/v) heat inactivated fetal bovine serum (ThermoFisher Scientific) and maintained at 37°C in a 95% $O_2$ + 5% $CO_2$ (carbogen) atmosphere. Cells were seeded on petri dishes 24 h prior to transfection. 2 µl Lipofectamine™2000 transfection agent (ThermoFisher Scientific) with 0.8 µg plasmid DNA was used to transfect cells. After 15 h-incubation with the DNA-lipid complex, cells were collected and re-plated on to glass coverslips. Whole-cell patch-clamp recordings started 3 h after coverslip plating.

REBL-PAT hiPSCs (gifted by Prof. Chris Denning, University of Nottingham) were differentiated into hiPSC-CMs as described [62]. Following differentiation, hiPSC-CMs were metabolically enriched using lactate [63]. Single cells were dissociated from monolayers 120 days post differentiation with 200 U/ml collagenase II (Worthington Biochemical Corp, Lakewood, NJ, USA) and plated on gelatin coated coverslips. Seeded cells were transduced with adenovirus on day 3 at multiplicity of infection of 5-10. Electrophysiogical recordings were performed on both untransduced and transduced cells on day 6.

During the recording, cells were bathed in extracellular solution containing (in mmol/l): 133 NaCl, 5.4 KCl, 1 $MgCl_2$, 2 $CaCl_2$, 10 HEPES, 11 glucose and pH adjusted to 7.4 with NaOH. The pipette solution contained (in mmol/l): 125 KAspartate, 10 KCl, 1 $MgCl_2$, 10 NaCl, 5 MgATP, 10 HEPES, and pH adjusted to 7.2 with KOH. For $I_{to,f}$ recordings, 200 µM $CdCl_2$ was applied. After a brief step from a holding potential of -80 mV to -40 mV to inactivate sodium channels, 500-ms voltage steps from -30 to +50 mV were used to elicit $I_{to}$ currents in hiPSC-CMs.

***Recombinant adenovirus production***

The bicistronic construct Kv4.3-mCherry-P2A-KChIP2.1was incorporated in adenovirus using the AdEasy™ adenoviral vector system (Addgene, USA) giving high transduction efficiency in the adult ventricular cardiomyocytes [64]. The transgenes were cloned into KpnI-HindIII sites of pShuttle-cytomegalovirus (CMV) vector (#16403, Addgene Watertown,Massachusetts, USA). Alternatively, cardiomyocyte-specific troponin T promoter with BsiWI and KpnI restriction sites at the 5' and 3' ends, respectively, was PCR amplified from a pAAV.cTNT vector (#86558, Addgene) and inserted upstream of the bicistronic transgenes in pShuttle plasmid (#16402, Addgene). Homologous recombinations were performed in bacterial AdEasy-1 cells (#16399, Addgene) and verified by restriction digestions. To produce adenovirus, HEK293 cells were transfected with the recombinant plasmid linearized by PmeI using Lipofectamine 2000 (ThermoFisher Scientific) in serum-diminished OPTI-MEM medium for 6 hours at 37°C. Virus was harvested 14 days later by 3x freezing-thawing the HEK293 cells and used for further virus amplification.

***Adult rabbit ventricular myocyte isolation, culture and adenovirus transduction***

Left ventricular (LV) cardiomyocytes were enzymatically isolated from the hearts of adult New Zealand rabbits as described previously [14]. Freshly isolated cardiac myocytes were first suspended in M199 supplemented with 5 mmol/l taurine, 5 mmol/l creatine, 0.01mmol/l ascorbic acid, 25 mmol/l HEPES, 0.2 % bovine serum albumin, 10 U/mL penicillin and 10 μg/mL streptomycin (all from Sigma Aldrich). After 2-h incubation at 37°C in 5% $CO_2$ cardiomyocytes were harvested and plated on glass coverslips coated with laminin (Sigma Aldrich) for 3 hours followed by infection with adenovirus carrying the target gene at multiplicity of infection (MOI) from 5-20. Myocytes were patch-clamped or immunostained 40 hours after viral transduction.

***Western Blotting***

Cells were washed three times with ice cold phosphate buffered saline and lysed in RIPA buffer (150 mmol/l NaCl, 50 mmol/l Tris-HCl, 1% Triton X-100, 1% sodium deoxycholate, 1 mmol/l EDTA, 2% sodium dodecyl sulfate (SDS), pH 7.4) containing additional cOmplete™, mini EDTA-free protease inhibitor mixture (MERCK, Sigma Aldrich, Dorset, UK) and a cocktail of phosphatase inhibitors (50

mmol/l NaF, 1 mmol/l $Na_3VO_4$ and 16 mmol/l $Na_4P_2O_7$) at 4°C. For detection of KChIP2.1 protein, cell lysate was mixed with 4x BoltTM LDS sample buffer (ThermoFisher Scientific), 10x BoltTM sample reducing agent (ThermoFisher Scientific) followed by complete protein denaturation by heating at 70°C for 10 min. For detection of Kv4.3 protein, samples were mixed with 2x Urea sample buffer containing 50 mmol/l Tris-HCl pH 6.8, 1.6% SDS, 7% glycerol, 8 mol/l urea, 4% β-mercaptoethanol and 0.016% bromophenol red followed by 30 min-incubation at room temperature. Proteins were separated by SDS-PAGE on Bolt$^{TM}$ 4-12% Bis-Tris Plus gels (ThermoFisher Scientific) and subsequently transferred to nitrocellulose membranes (Amersham, Buckinghamshire, UK). Total protein transferred to the membrane were stained by SYPRO® Ruby (ThermoFisher Scientific) as a loading control prior to membrane blocking in Tris-buffered solution containing 5% dry milk and 0.1% Tween 20. Blots were then incubated with 1:2000 rabbit anti-Kv4.3 or 1:2000 rabbit anti-KChIP2  primary antibodies (both Alomone Labs, Jerusalem, Israel) overnight at 4°C followed by 1-hour exposure to 1:5000 (cardiomyocyte experiments) or 1:8000 horseradish peroxidase (HRP)-conjugated anti-rabbit secondary antibody (Promega, Southampton, UK) at room temperature. Finally, immunolabelled membranes were detected using Amersham ECL Western Blotting detection system (GE Healthcare Life Sciences, Little Chalfont, UK). SuperSignal West Femto Maximum Sensitivity Substrate (ThermoFisher Scientific) was used to detect proteins on the membrane.

***Myocyte contraction measurements***

The myocytes were field stimulated to contract at a frequency of 1 Hz using a pair of platinum wires placed on opposite sides of the recording chamber and connected to a Grass Stimulator (Grass, ). Fractional shortening was determined as %S= ΔL/L0 from transmitted light images obtained during laser scanning.

**Supplementary References**

[62] D. Mosqueira, I. Mannhardt, J.R. Bhagwan, K. Lis-Slimak, P. Katili, E. Scott, et al., CRISPR/Cas9 editing in human pluripotent stem cell-cardiomyocytes highlights

arrhythmias, hypocontractility, and energy depletion as potential therapeutic targets for hypertrophic cardiomyopathy, European Heart Journal. 39 (2018) 3879–3892. doi:10.1093/eurheartj/ehy249.

[63] S. Tohyama, F. Hattori, M. Sano, T. Hishiki, Y. Nagahata, T. Matsuura, et al., Distinct metabolic flow enables large-scale purification of mouse and human pluripotent stem cell-derived cardiomyocytes, Cell Stem Cell. 12 (2013) 127–137. doi:10.1016/j.stem.2012.09.013.

[64] J. Luo, Z.-L. Deng, X. Luo, N. Tang, W.-X. Song, J. Chen, et al., A protocol for rapid generation of recombinant adenoviruses using the AdEasy system, Nature Protocols. 2 (2007) 1236–1247. doi:10.1038/nprot.2007.135.
